# Exploring the Interplay of Stress, Sleep Quality, and Lifestyle Factors in Bruxism Among Undergraduate Students in Saudi Arabia: A Cross-Sectional Study

**DOI:** 10.3390/healthcare14193312

**Published:** 2026-10-04

**Authors:** Reem M. Alwhaibi, Remaz Almawsh, Raghad Almutairi, Dalaa Alowaishek, Dhai Aldubayan

**Affiliations:** Department of Rehabilitation Sciences, College of Health and Rehabilitation Sciences, Princess Nourah Bint Abdulrahman University, P.O. Box 84428, Riyadh 11671, Saudi Arabia; 443000438@pnu.edu.sa (R.A.); 443009117@pnu.edu.sa (R.A.); 443000303@pnu.edu.sa (D.A.); 443000274@pnu.edu.sa (D.A.)

**Keywords:** bruxism, stress, sleep quality, university students, lifestyle factors, Saudi Arabia, psychological health, oral parafunction

## Abstract

**Background**: Bruxism, a repetitive clenching or grinding of teeth, is a prevalent yet under-recognized issue among university students. Its etiology is multifactorial, often involving psychological stress, poor sleep quality, and unhealthy lifestyle behaviors. Despite increasing evidence linking these variables, limited research has examined these associations within Middle Eastern populations. **Objectives**: This study aimed to assess the proportion screening positive for bruxism risk among Saudi undergraduate students and to investigate its associations with perceived stress, sleep quality, and lifestyle factors. **Methods**: A cross-sectional survey was conducted among 335 Saudi undergraduate students aged 18–25 years. Data were collected using the 25-item BRUXiq self-assessment questionnaire, the 11-item Stress Self-Assessment Questionnaire, the Pittsburgh Sleep Quality Index (PSQI), and demographic and behavioral questions. Descriptive statistics, Chi-square tests, Mann–Whitney U tests, Spearman’s correlations, and binary logistic regression were performed. **Results**: A total of 198 participants (59.1%) screened positive for bruxism risk according to the BRUXiq criterion, whereas 137 (40.9%) screened negative. Participants with a positive BRUXiq screen exhibited significantly higher stress scores (*p* < 0.001) and poorer sleep quality (*p* < 0.001). Multivariable logistic regression showed that higher stress scores (OR = 1.123, 95% CI [1.08, 1.17]) and poorer sleep quality (OR = 1.188, 95% CI [1.07, 1.31]) were independently associated with greater odds of a positive BRUXiq screen. No significant associations were found for smoking, stimulant use, physical activity, or academic year. **Conclusions**: A positive BRUXiq screening result was common among the participating students and was associated with higher stress and poorer sleep quality. Because bruxism was assessed using a self-report screening questionnaire, the findings should not be interpreted as the prevalence of clinically confirmed bruxism. These findings underscore the importance of psychological support services and sleep hygiene education within university health programs as potential supportive strategies that require further evaluation.

## 1. Introduction

Bruxism, defined as the repetitive and involuntary grinding or clenching of teeth, is a prevalent oral parafunctional activity that can occur during wakefulness or sleep. While bruxism can affect individuals across all age groups, university students have emerged as a particularly vulnerable population due to the psychological and physiological demands of academic life [1,2]. Recent global estimates indicate an overall bruxism prevalence of 22.22%, with prevalence estimates of 21% for sleep bruxism and 23% for awake bruxism. The global co-occurrence of bruxism and temporomandibular disorders (TMDs) has been estimated at approximately 17%, with substantial variation across continents [3]. Bruxism may be associated with oral and musculoskeletal consequences, including tooth wear, tooth mobility, and temporomandibular symptoms, and frequently co-occurs with psychological distress and sleep disturbances [4,5].

Bruxism has been associated with multiple psychological, sleep-related, and behavioral factors, including psychological stress, sleep disturbances, caffeine use, and smoking [6,7].

Psychological stress has been widely associated with both sleep and awake bruxism. Ref. [8] reported that higher levels of perceived stress were significantly correlated with increased bruxism activity, particularly in student populations. Similarly, Ref. [9] found that nearly 39% of students experiencing stress displayed bruxism symptoms, supporting an association between psychological stress and bruxism-related symptoms in student populations.

Poor sleep quality has also been associated with bruxism. Students with irregular sleep schedules, late-night study routines, and limited sleep duration have shown a higher prevalence of bruxism-related behaviors [10,11]. Lavigne et al. [12] described physiological relationships between sleep processes and sleep bruxism, including mechanisms involving sleep arousal and masticatory muscle activity.

However, the direction of the relationship between sleep disturbances and bruxism cannot be established from cross-sectional evidence.

Despite this, findings are sometimes inconsistent. For instance, Ref. [13] found no significant correlation between subjective sleep quality and sleep bruxism, suggesting that the association between subjective sleep quality and bruxism may vary across populations and assessment methods.

Lifestyle behaviors have also been examined in relation to bruxism. The consumption of psychoactive substances, particularly caffeine, alcohol, and tobacco, has been positively associated with bruxism in student populations [11].

Although previous studies have examined psychological, sleep-related, and lifestyle correlates of bruxism, evidence integrating these factors within Saudi undergraduate populations remains limited. Existing Saudi evidence has also tended to focus on specific student groups or individual correlates rather than examining perceived stress, sleep quality, and selected lifestyle behaviors within the same undergraduate sample. Examining these variables in a Saudi university population may therefore provide context-specific evidence regarding factors associated with self-reported bruxism without assuming that the observed relationships are causal.

Accordingly, this study aimed to determine the prevalence of questionnaire-defined, self-reported bruxism among undergraduate students in Saudi Arabia and to examine its associations with stress, sleep quality, and selected behavioral factors, including smoking and physical activity.

By examining these variables within the same study population, this study may provide context-specific evidence to inform future research and student health initiatives in Saudi Arabia.

## 2. Methodology

### 2.1. Study Design

This study employed a cross-sectional design to investigate the proportion screening positive for bruxism risk and its associations with psychological stress, sleep quality, and lifestyle behaviors among undergraduate students in Saudi Arabia. Data collection was conducted over a four-month period, from 2 January to 29 April 2025.

Ethical approval was obtained from the Institutional Review Board of Princess Nourah Bint Abdulrahman University (IRB # 24-0491) on 25 December 2024. Participant recruitment and data collection commenced only after ethical approval had been obtained. All methods were performed in accordance with the relevant guidelines and regulations, including the principles of the Declaration of Helsinki. Participation was voluntary and anonymous. Before accessing the questionnaire, all participants were required to read an informed consent statement outlining the study’s objectives, their rights as participants, and data confidentiality protocols. Electronic informed consent was obtained prior to eligibility screening and survey participation. Data were stored securely in encrypted files, accessible only to the research team.

### 2.2. Participants

Eligibility was determined using a self-reported screening form completed before access to the main questionnaire. For the analytical sample, participants were excluded if they did not provide informed consent, reported a diagnosed temporomandibular joint (TMJ) disorder, reported a dental condition that could affect teeth grinding, or reported current use of antidepressants or other medications affecting teeth grinding. Eligibility information was obtained through self-reported screening questions. According to the Saudi Ministry of Education [14], the university student population in Saudi Arabia was approximately 1,297,426. The minimum required sample size was calculated for estimation of a population proportion using an expected bruxism prevalence of 20% [15], a 95% confidence level, a 5% absolute margin of error, and the reported population size. Based on these parameters, the minimum required sample size was approximately 246 participants. A recruitment target of 400 participants was established to account for potential exclusions and non-response and to ensure an adequate final analytical sample.

A non-probability convenience sampling approach was used to recruit students from different academic disciplines and year levels across multiple institutions. Students from both health-related and non-health-related academic programs were approached to increase representation across disciplines. The implications of convenience sampling for selection bias and generalizability are addressed in the Section 4.1.

### 2.3. Data Collection Tool

This study used a structured, self-administered online questionnaire. Following electronic consent and eligibility screening, the questionnaire comprised four main components: (1) demographic and behavioral characteristics, (2) the 25-item BRUXiq self-assessment questionnaire, (3) an 11-item Stress Self-Assessment Questionnaire, and (4) the Pittsburgh Sleep Quality Index (PSQI). The demographic and behavioral component included nine questions addressing age, sex, academic year, field of study, marital status, residential status, monthly family income, smoking status, and daily physical activity level. Bruxism-related behaviors and symptoms were assessed using the 25-item BRUXiq self-assessment questionnaire described by Chanteux et al. [16]. The instrument assesses self-reported awake and sleep-related tooth grinding and jaw clenching, parafunctional oral behaviors, and associated oral, jaw, sleep-related, and morning symptoms. Each item was rated on a four-point response scale: 0 = No, 1 = Yes, slightly, 2 = Yes, moderately, and 3 = Yes, a lot. Item scores were summed to produce a total BRUXiq score ranging from 0 to 75. In the present study, BRUXiq scores <15 were classified as a negative screen, whereas scores ≥15 were classified as a positive screen for bruxism risk. This operational classification was based on the scoring framework reported by Chanteux et al. [16]. The BRUXiq was treated as a self-report screening measure rather than a clinical diagnostic assessment. No separate bruxism severity categories were calculated. The stress component was assessed using the 11-item Stress Self-Assessment Questionnaire described by Chanteux et al. [16]. The questionnaire assesses stress- and anxiety-related emotional, physical, and behavioral manifestations, including emotional sensitivity, irritability, perfectionistic tendencies, autonomic symptoms, muscular tension, sleep problems, worry, somatic symptoms, fatigue, health-related symptoms, and the use of substances or medications for stimulation or calming. Each item was scored on a seven-point scale from 0 (not at all) to 6 (extremely), producing a total score ranging from 0 to 66. According to the published scoring system, scores were divided into four ranges: <15, 15–32, 33–45, and 46–66. For descriptive reporting in the present study, these ranges were operationally categorized as no stress, low stress, moderate stress, and high stress, respectively. Sleep quality was assessed using the Pittsburgh Sleep Quality Index (PSQI), a standardized self-report instrument assessing sleep quality over the previous month. The PSQI yields seven component scores: subjective sleep quality, sleep latency, sleep duration, habitual sleep efficiency, sleep disturbances, use of sleep medication, and daytime dysfunction. Each component is scored from 0 to 3, and the seven components are summed to obtain a global PSQI score ranging from 0 to 21, with higher scores indicating poorer sleep quality. A global PSQI score ≤5 was classified as good sleep quality, whereas a score >5 was classified as poor sleep quality, in accordance with the standard PSQI scoring procedure. The Arabic version of the PSQI has been validated in multiple studies, with reported Cronbach’s alpha values exceeding 0.70 [17], indicating acceptable reliability. The Arabic PSQI was scored according to the established scoring procedure.

### 2.4. Validation and Pilot Testing of Data Collection Tool

Prior to the main data collection, the questionnaire underwent pilot testing with 20 undergraduate students representing different academic disciplines and study levels. The pilot assessment evaluated item clarity, language comprehensibility, questionnaire flow, response options, and completion time.

Feedback was used to refine the presentation and clarity of the Arabic questionnaire. The questionnaire was also reviewed by experts in dentistry, clinical psychology, and epidemiology for content relevance, clarity, and cultural appropriateness. The BRUXiq and Stress Self-Assessment Questionnaire were based on the previously published instruments reported by Chanteux et al. [16]. The PSQI was administered using its established Arabic version [17]. Internal consistency in the present sample was α = 0.825 for the 25-item BRUXiq, α = 0.827 for the 11-item Stress Self-Assessment Questionnaire, and α = 0.55 for the PSQI.

### 2.5. Procedure

Participants were recruited through multiple digital channels, including university mailing lists, student forums, and widely used social media platforms such as WhatsApp, Telegram, and Twitter. This multi-channel approach was intended to maximize participation and ensure outreach across a diverse range of academic programs and institutions.

Upon accessing the survey link, participants first encountered a standardized introductory screen that provided detailed information about the study’s purpose, the voluntary nature of participation, data confidentiality, and anonymity assurances. Participants were required to indicate their agreement with the informed consent statement electronically before proceeding further.

Following consent, an eligibility screening form was presented to determine compliance with the study’s inclusion and exclusion criteria. Eligible participants were then directed to the full questionnaire, which required approximately 10–15 min to complete.

To improve response rates, automated reminder messages were distributed biweekly via institutional email systems and student community channels. All survey responses were automatically recorded and stored in real time. Data were secured using encrypted, password-protected systems with access restricted to the principal investigator and authorized research team members only.

### 2.6. Data Analysis

All statistical analyses were conducted using IBM SPSS Statistics version 27.0. Descriptive statistics, including means, standard deviations, frequencies, and percentages, were calculated to summarize participant characteristics and responses to the bruxism, stress, sleep quality, and lifestyle-related items.

BRUXiq status was classified according to the predefined cutoff described above. Stress scores were analyzed as continuous scores in the principal continuous and multivariable analyses; for descriptive and χ^2^ analyses, scores were operationally categorized as <15, 15–32, 33–45, and 46–66, corresponding to no, low, moderate, and high stress, respectively. PSQI global scores were analyzed continuously and were also categorized as good sleep quality (≤5) or poor sleep quality (>5) for categorical analyses. Continuous scores were retained in the principal analyses to avoid unnecessary loss of information from categorization.

Effect-size measures were reported alongside inferential tests. For χ^2^ analyses, φ was used for 2 × 2 contingency tables and Cramér’s V for larger contingency tables. Rank-biserial correlation was used as the effect-size measure for Mann–Whitney U comparisons, Spearman’s ρ was reported for correlation analyses, and odds ratios (ORs) with 95% confidence intervals (CIs) were reported for logistic regression. Where available, 95% CIs were also reported for effect-size estimates. Correlation magnitudes were interpreted using the dentistry-specific thresholds proposed by Zieliński and Więckiewicz [18], with values of approximately 0.20, 0.40, and 0.70 interpreted as small, medium, and large effects, respectively. Normality of the data was assessed using the Kolmogorov–Smirnov test. Given the non-normal distribution of most scale variables (e.g., stress and sleep quality scores), non-parametric tests were employed for group comparisons. Specifically, Chi-square tests were used to examine associations between BRUXiq screening status (negative/positive) and categorical variables such as stress level, sleep quality category, smoking status, and academic year. Mann–Whitney U tests were applied to compare stress and sleep quality scores between participants with positive and negative BRUXiq screens. Spearman’s rank-order correlation was used to assess the strength and direction of associations between BRUXiq total scores, stress scores, and sleep quality scores.

Binary logistic regression was used to examine factors independently associated with a positive BRUXiq screen. BRUXiq screening status was the dependent variable and was coded as 1 = negative screen and 2 = positive screen. Stress score, PSQI global score, smoking status, stimulant use, physical activity level, and academic year were entered as independent variables. For categorical variables, the last category was used as the reference category: non-smoking for smoking status, high physical activity for physical activity level, and sixth year for academic year. Multicollinearity was assessed using variance inflation factors (VIFs), and linearity of the continuous variables with the logit was assessed using the Box–Tidwell procedure. Independence of observations was maintained because each participant contributed one observation. Model fit was evaluated using the Hosmer–Lemeshow goodness-of-fit test and Nagelkerke R^2^. Odds ratios (ORs) with 95% confidence intervals (CIs) were reported. Statistical significance was set at *p* < 0.05.

## 3. Results

**Figure 1:**
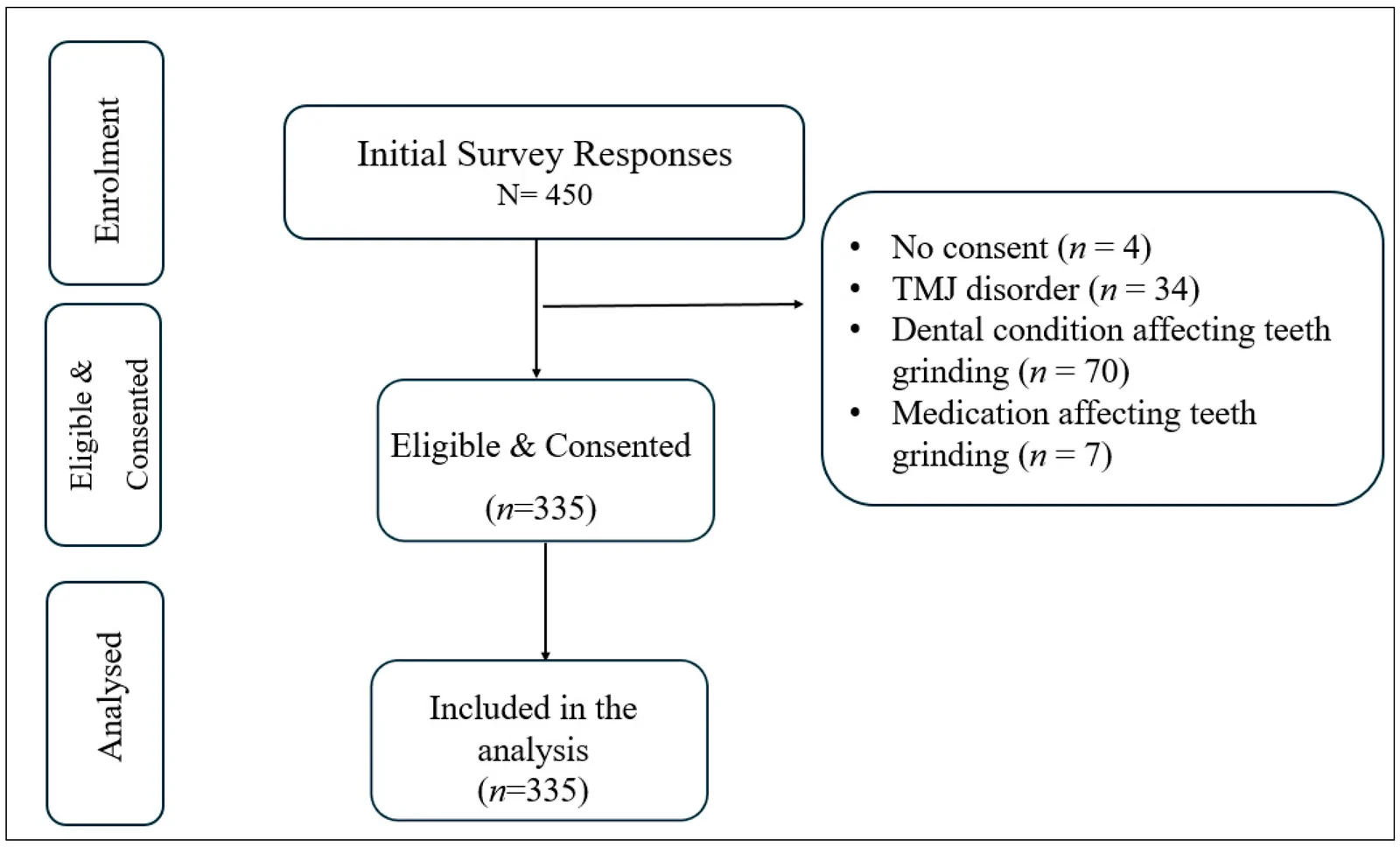
Flow chart of participants’ recruitment.

A total of 450 survey responses were initially obtained. Four respondents were excluded because they did not provide informed consent. Among the remaining respondents, 34 were excluded because they reported a diagnosed temporomandibular joint disorder, 70 because they reported a dental condition that could affect teeth grinding, and 7 because they reported current use of antidepressants or other medications affecting teeth grinding. After sequential application of these exclusion criteria, 335 participants remained and were included in the final analysis (Figure 1).

### 3.1. Demographic Characteristics

The majority of participants (85.7%) were aged between 18 and 22 years, and 74% were females. Most students were in their first academic year (35.5%) or second (20.9%). Participants represented a range of academic disciplines, with 31.6% enrolled in health and medical sciences and 20.6% in engineering and technology. The majority of participants (97%) were single, 89.3% lived with their families, and 48.4% had a monthly family income of more than 10,000 SR. The majority (93.4%) were non-smokers and reported moderate (53.1%) or low (38.8%) physical activity level (Table 1).

### 3.2. BRUXiq Screening Results and Item Scores

Based on the screening, 198 participants (59.1%) screened positive for bruxism risk, whereas 137 (40.9%) screened negative. Screening status was determined using a predefined scoring threshold across 25 bruxism-related items. The overall mean BRUXiq score among participants was 18.24 (SD = 10.08). Among the core items, the highest mean scores were observed for awake jaw clenching (M = 0.82, SD = 0.94), jaw or muscle fatigue upon waking (M = 0.49, SD = 0.89), and reports of grinding or clenching during sleep (M = 0.57, SD = 0.88). Additionally, participants reported symptoms often associated with bruxism and poor sleep quality, including daytime tiredness after waking (M = 1.62, SD = 1.04), emotional sensitivity (M = 1.49, SD = 1.12), and cheek or lip biting (M = 1.06, SD = 1.06). These findings suggest a multifaceted symptom profile that includes both physical and psychosomatic features (Table 2).

### 3.3. BRUXiq Screening Status by Age, Gender, and Academic Year

Bruxism was slightly more prevalent in students aged 23–25 years (63%) compared to those aged 18–22 years (59%). It was more common among females (62%) than males (51%). By academic year, the highest prevalence was observed in fifth-year students (73%), followed by fourth-year (66%) and sixth-year students (60%) (Table 3).

### 3.4. Stress Levels

The mean total stress score was 18.37 (SD = 11.06). The highest mean item scores were observed for feeling worried (M = 2.69, SD = 1.96), feeling tired (M = 2.49, SD = 1.87), and being quick to anger or irritability (M = 2.42, SD = 1.68). The lowest mean scores were observed for the use of products or medications for stimulation or calming (M = 0.37, SD = 1.04) and serious health-related symptoms (M = 0.46, SD = 1.22) (Table 4).

Based on the predefined stress-score categories, 145 participants (43.3%) were classified in the no-stress category, 150 (44.8%) in the low-stress category, 35 (10.4%) in the moderate-stress category, and 5 (1.5%) in the high-stress category. Overall, 88.1% of participants were classified as having no or low stress (Table 5).

### 3.5. Sleep Quality

The mean global PSQI score was 7.62 (SD = 3.11), suggesting poor sleep quality. The highest component scores were for sleep latency (M = 1.64, SD = 0.97) and daytime dysfunction (M = 1.47, SD = 0.91), while the lowest was for sleep medication use (M = 0.21, SD = 0.64) (Table 6). A total of 73.7% of students had poor sleep quality, while 26.3% had good sleep quality (Table 7).

### 3.6. Associations Between Bruxism, Stress, and Sleep Quality

Chi-square tests showed a significant association between BRUXiq screening status and stress level (χ^2^ = 51.217, *p* < 0.001; Cramér’s V = 0.391) and between BRUXiq screening status and sleep quality (χ^2^ = 18.453, *p* < 0.001; φ = 0.235). No significant associations were observed between BRUXiq screening status and academic year (*p* = 0.343) or smoking status (*p* = 0.179) (Table 8).

For the stress categories, the proportions screening positive were 40.0% in the no-stress group (58/145; 95% CI, 32.4–48.1%), 67.3% in the low-stress group (101/150; 95% CI, 59.5–74.3%), 100.0% in the moderate-stress group (35/35; 95% CI, 90.1–100.0%), and 80.0% in the high-stress group (4/5; 95% CI, 37.6–96.4%). Estimates for the moderate- and particularly the high-stress groups should be interpreted cautiously because of the small subgroup sizes.

### 3.7. Comparison of Stress and Sleep Quality by Bruxism Status

Normality tests (Kolmogorov–Smirnov) indicated non-normal distribution for most variables, justifying the use of non-parametric tests (Table 9). Mann–Whitney U tests revealed that students with bruxism had significantly higher stress scores (U = 6223.5, Z = −8.426, *p* < 0.001), with medium effect size (r_rb = 0.541, 95% CI [0.438,0.636]) and poorer sleep quality scores (U = 7727, Z = −6.731, *p* < 0.001) compared to those without bruxism, with a moderate effect size (r_rb = 0.430, 95% CI [0.315,0.534]) (Table 10).

Mann–Whitney U tests showed that participants with a positive BRUXiq screen had significantly higher stress scores than those with a negative screen (U = 6264.0, Z = −8.379, *p* < 0.001), with a moderate rank-biserial correlation (r_rb = 0.538, 95% CI [0.437, 0.634]). Participants with a positive BRUXiq screen also had poorer sleep-quality scores (U = 7727, Z = −6.731, *p* < 0.001; r_rb = 0.430, 95% CI [0.315, 0.534]) (Table 10).

### 3.8. Correlation Analysis Between Bruxism Score and Stress/Sleep Scores

Spearman’s correlation analysis showed significant moderate positive correlations between BRUXiq totalscores and stress score (ρ = 0.579, *p* < 0.001, 95% CI [0.503, 0.646]) and between BRUXiq totalscores and sleep quality (ρ = 0.408, *p* < 0.001, 95% CI [0.312, 0.496]) (Table 11).

### 3.9. Factors Associated with a Positive BRUXiq Screen

Regression assumptions were examined before model estimation. No problematic multicollinearity was identified (VIF = 1.24), and the Box–Tidwell interaction terms were not statistically significant (*p* > 0.05), supporting linearity of the continuous predictors with the logit.

Logistic regression showed that stress and sleep quality were significantly associated with a positive BRUXiq screen. The model had a Nagelkerke R^2^ of 0.362, and the Hosmer–Lemeshow test indicated no evidence of poor model fit (χ^2^ = 8.194, *p* = 0.415) (Table 12).

Each one-unit increase in stress score was associated with 12.3% higher odds of a positive BRUXiq screen (OR = 1.123, 95% CI [1.083, 1.165], *p* < 0.001). Each one-unit increase in PSQI global score, indicating poorer sleep quality, was associated with 18.8% higher odds of a positive BRUXiq screen (OR = 1.188, 95% CI [1.074, 1.314], *p* < 0.001).

### 3.10. Sleep Partner Observations

Of the 335 participants, 174 (51.9%) reported having no bed partner or roommate. Partner-observed sleep behaviors were therefore summarized for the 161 participants with a roommate or bed partner. Among these 161 participants, 117 (73%) reported no loud snoring, 126 (78%) reported no observed breathing pauses, and 74 (46%) reported no leg twitching or jerking during sleep (Table 13).

### 3.11. Sleep Disturbances (Open-Ended Responses)

Sixty participants provided open-ended descriptions of sleep disturbances. Because a single response could contain more than one sleep-related theme, multiple coding was permitted, yielding 78 coded themes. The most commonly reported themes were insomnia (16.7%), nightmares or disturbing dreams (15.0%), shortness of breath or difficulty breathing during sleep (13.3%), and anxiety, overthinking, or stress before or during sleep (13.3%) (Table 14).

## 4. Discussion

The present study identified a relatively high proportion of Saudi undergraduate students who screened positive for bruxism using the BRUXiq questionnaire. This estimate is higher than many prevalence estimates reported in general adult populations [10,18] and the recent global pooled prevalence estimate of approximately 22% [3]. However, relatively high frequencies of bruxism have also been reported in university-student populations [11,15]. Direct comparisons between studies should nevertheless be made cautiously because prevalence estimates vary substantially according to the population studied and, importantly, the method used to identify bruxism [10,19]. In the present study, classification was based on a self-report screening questionnaire rather than clinical examination or instrumental assessment; therefore, the observed prevalence should be interpreted as questionnaire-defined bruxism risk rather than clinically confirmed bruxism.

This distinction is particularly relevant because several frequently endorsed BRUXiq items in the present sample reflected relatively non-specific experiences, such as tiredness, emotional sensitivity, or perceived stress, whereas more characteristic manifestations, including observed tooth grinding and some morning jaw symptoms, were less frequently endorsed. Consequently, the relatively high proportion of positive screens may partly reflect the broad range of behaviors and symptoms included in the questionnaire. Previous clinical reviews have emphasized that the assessment of bruxism depends on the method used and that self-report alone cannot provide the same level of confirmation as clinical or instrumental assessment [12,19]. Thus, the BRUXiq findings are useful for identifying individuals with possible bruxism-related behaviors or symptoms, but they should not be interpreted as definitive diagnoses. Accordingly, the BRUXiq should be interpreted as a screening measure for possible bruxism-related behaviors and symptoms rather than as a clinical diagnostic tool.

Although descriptive differences in positive BRUXiq screening were observed according to sex and academic year, these patterns should be interpreted cautiously. The present study did not directly investigate mechanisms that might explain sex differences, and academic year was not independently associated with BRUXiq screening status in the adjusted model. Accordingly, explanations based on presumed differences in stress exposure, symptom awareness, graduation pressure, or accumulated academic burden cannot be established from these data. Similarly, because field of study was not formally evaluated as an independent factor, the findings do not support conclusions that students in particular programs, such as medicine or engineering, are more susceptible to bruxism. This more cautious interpretation is also consistent with the considerable heterogeneity in demographic patterns reported across bruxism studies [20].

The association between stress and questionnaire-defined bruxism is consistent with a substantial body of previous evidence. Cavallo et al. reported an association between perceived stress and bruxism among university students [8], while systematic reviews and meta-analyses have also demonstrated associations between stress symptoms and bruxism [7,20]. Similar relationships have been reported among Brazilian university students [21] and in studies examining psychological factors among university populations [15]. These studies support the interpretation that psychological stress and bruxism-related behaviors frequently co-occur, although they do not establish that stress causes bruxism. In the present study, particular caution is required when interpreting the very high proportion of positive BRUXiq screens in the moderate- and high-stress categories because these categories contained comparatively few participants.

Several biological and behavioral mechanisms linking psychological stress with bruxism have been proposed in the literature [7,20]; however, these mechanisms were not assessed in the present study and therefore cannot be inferred from the current findings.

The observed association between poorer sleep quality and positive BRUXiq screening is also broadly consistent with previous research. Yıldırım et al. reported associations between self-reported bruxism, sleep quality, and psychological status among dental students [22], while other reviews have described relationships between sleep bruxism and sleep-related disturbances [6,23]. Physiological research has also linked sleep bruxism episodes with sleep arousal processes and masticatory muscle activity [12]. Nevertheless, the evidence is not entirely consistent. A systematic review by Yanez-Regonesi et al. found variability in the relationship between sleep quality and different levels of sleep-bruxism assessment [13]. The present findings therefore add to evidence of an association between poorer subjective sleep quality and bruxism-related behaviors but do not establish the direction of that relationship. Because the present design was cross-sectional, it cannot determine whether sleep disturbance precedes bruxism-related behaviors, follows them, or whether both are associated with other shared factors.

The magnitude of the observed associations should also be considered. The correlations of BRUXiq scores with stress and sleep quality were of moderate magnitude according to the effect-size thresholds applied in the analysis. Each one-point increase in stress score was associated with higher odds of a positive BRUXiq screen (OR = 1.123, 95% CI [1.083, 1.165]), and each one-point increase in PSQI score was similarly associated with higher odds (OR = 1.188, 95% CI [1.074, 1.314]). These estimates represent changes in odds per one-point increase in the respective scales and should not be interpreted as equivalent percentage changes in the probability of bruxism. Although these adjusted associations support the relevance of stress and sleep quality within this sample, the cross-sectional design does not establish that either factor precedes or causes bruxism-related behaviors.

Smoking, stimulant use, physical activity, and academic year were not independently associated with positive BRUXiq screening in the adjusted analysis. This should not be interpreted as evidence that these factors have “limited” or “indirect” effects. Previous research has examined behavioral factors such as smoking and psychoactive-substance use in relation to bruxism, with some studies reporting associations [11,24,25]. The absence of a statistically significant association in the present study may reflect differences in population characteristics, measurement, or statistical precision. In particular, smokers represented only a small proportion of the sample, which limits the precision with which an association with smoking can be evaluated.

Overall, the findings are consistent with previous evidence demonstrating associations of psychological stress and sleep-related factors with bruxism-related behaviors in university and adult populations [7,8,20,21,22]. The present study extends this evidence to Saudi undergraduate students; however, interpretation should remain within the limits of questionnaire-based assessment and the cross-sectional design. Future studies using clinical or instrumental assessment methods are needed to confirm these associations and more accurately characterize bruxism in this population.

### 4.1. Limitations

This study has several limitations that should be acknowledged. First, the use of self-reported questionnaires may introduce potential recall bias, social desirability bias, and subjective interpretation of questionnaire items. These factors may influence the accuracy of responses related to bruxism behaviors, stress levels, and sleep quality. In addition, bruxism was assessed using the BRUXiq self-report screening questionnaire without objective or clinical verification. Therefore, some participants may have been misclassified, and the estimated prevalence of positive BRUXiq screening should not be interpreted as the prevalence of clinically confirmed bruxism.

Second, the cross-sectional design precludes determination of temporal relationships and causal direction between bruxism-related behaviors, stress, sleep quality, and the other factors examined.

Third, participants were recruited using convenience sampling, which may have introduced selection bias. Consequently, the sample may not be fully representative of the wider population of university students in Saudi Arabia. Generalizability is therefore limited not only to university populations in other countries but also to the broader population of Saudi university students.

Fourth, women represented 74% of the sample, resulting in a marked sex imbalance. Accordingly, the findings may disproportionately reflect the experiences of female students and should be generalized cautiously to more sex-balanced university populations. Although students from different academic disciplines were included, this diversity does not eliminate the limitations associated with non-probability sampling and the demographic imbalance of the sample.

### 4.2. Recommendation

The findings support the potential value of increasing awareness of bruxism-related behaviors and symptoms among university students and encouraging students with persistent symptoms to seek appropriate professional assessment. Universities may consider educational activities addressing bruxism, stress awareness, and healthy sleep practices, as well as facilitating access to dental or maxillofacial assessment for students reporting relevant symptoms.

Given the observed associations between positive BRUXiq screening, stress, and poorer sleep quality, psychological support and sleep-health initiatives may also represent relevant areas for future university-based interventions. However, the present cross-sectional study did not evaluate the effectiveness of specific interventions, such as stress-management programs, relaxation techniques, sleep treatment, reductions in academic workload, or family involvement. These approaches should therefore be considered potential avenues for future intervention studies rather than direct recommendations arising from the present findings.

### 4.3. Future Directions

Future research should prioritize the use of more rigorous and clinically validated criteria for assessing bruxism, including objective or instrument-based methods where feasible, to improve diagnostic accuracy and reduce potential misclassification. Where the aim is to distinguish between sleep bruxism and awake bruxism, these conditions should be assessed and analyzed separately using appropriate criteria for each condition.

Longitudinal studies are also needed to clarify the temporal relationships among stress, sleep disturbances, and bruxism-related behaviors. Incorporating objective assessment methods, such as electromyography or polysomnography where appropriate, may provide more accurate estimates and strengthen the interpretation of future findings.

Further studies may also examine psychological factors such as coping style, emotional regulation, and resilience, and comparative research across universities and different settings may help improve the generalizability of the evidence.

## 5. Conclusions

A substantial proportion of participants screened positive for bruxism risk using the BRUXiq questionnaire. Higher stress and poorer sleep quality were independently associated with greater odds of a positive BRUXiq screen, while smoking, physical activity, and academic year were not independently associated in the adjusted model. Because the study was cross-sectional and bruxism was identified using a self-report screening questionnaire, the findings do not establish causality and should not be interpreted as clinically confirmed bruxism prevalence.

## Figures and Tables

**Table 1:** Demographic characteristics of study participants (*n* = 335).

Characteristic	Categories	*n*	%
Age	From 18 to 22 years	287	85.7
	From 23 to 25 years	48	14.3
Sex	Male	87	26
	Female	248	74
Year of study	First	119	35.5
	Second	70	20.9
	Third	45	13.4
	Fourth	61	18.2
	Fifth	15	4.5
	Sixth	25	7.5
Specialization	art, design, media	3	0.9
	Business, Economics, and Management	43	12.8
	education	10	3
	Engineering and technology	69	20.6
	Health and medical sciences	106	31.6
	Humanities and social sciences	38	11.3
	Law and legal studies	34	10.1
	Natural and applied sciences	25	7.5
	other	7	2.1
Marital status	Single	325	97
	Married	9	2.7
	Engaged	1	0.3
Residential status	I live alone	13	3.9
	I live with my family	299	89.3
	I live in the student residence	14	4.2
	I live with colleagues/friends	7	2.1
	I live with my sister/brother	1	0.3
	With my uncle and his family	1	0.3
Monthly family income	Less than 5000	83	24.8
	Between 5000 and 10,000	90	26.9
	More than 10,000	162	48.4
Smoking	No	313	93.4
	Yes	22	6.6
daily physical activity level	Low	130	38.8
	Moderate	178	53.1
	High	27	8.1

**Table 2:** Bruxism Assessment among Undergraduate Students in Saudi Arabia (*n* = 335).

No	Items	Mean	SD
1	Do you think you grind your teeth while sleeping	0.57	0.88
2	Do you grind your teeth while awake	0.42	0.74
3	Do you clench your jaw muscles or teeth while awake	0.82	0.94
4	Do you move your teeth by clenching them while awake	0.41	0.80
5	Do you bite your nails	0.78	1.09
6	Do you chew gum frequently	1.01	1.04
7	Do you chew your cheeks, lips or other things	1.06	1.06
8	Do you press your tongue or lips against your teeth	0.78	1.02
9	Do you breathe through your mouth a lot	0.73	0.95
10	Do you wake up at night and realize you’ve been clenching your teeth	0.48	0.85
11	Do you feel tired in your jaw muscles when you wake up	0.49	0.89
12	Do you feel pain or numbness in your teeth, like anesthesia, when you wake up	0.21	0.62
13	Are you having trouble sleeping	1.06	1.06
14	Do you think you snore while sleeping	0.43	0.77
15	Do you wake up with a dry mouth	0.96	1.05
16	Do you feel tired or sleepy after waking up	1.62	1.04
17	Do you find your social environment stressful	1.20	1.09
18	Do you consider yourself emotionally sensitive	1.49	1.12
19	Do you frequently consume stimulants such as tobacco or coffee	1.18	1.21
20	Do you suffer from problems with acidity in the mouth, such as reflux or nausea	0.54	0.92
21	Are your teeth generally sensitive	0.81	0.91
22	Do you wake up in the morning with a headache	0.60	0.83
23	Do you have nervous disorders	0.28	0.64
24	Do you have difficulty opening your mouth in the morning	0.16	0.47
25	Has anyone ever heard that you grind your teeth while sleeping	0.14	0.49
	Total (sum score)	18.24	10.08

**Table 3:** Distribution of BRUXiq screening status according to gender, age, and academic year (*n* = 335).

Characteristic	Bruxism	Negative BRUXiq Screen		Positive BRUXiq Screen	
	Total	137	40.9%	198	59.1%
Age	From 18 to 22 years	119	41%	168	59%
	From 23 to 25 years	18	38%	30	63%
Sex	Male	43	49%	44	51%
	Female	94	38%	154	62%
Year of study	First	46	39%	73	61%
	Second	33	47%	37	53%
	Third	23	51%	22	49%
	Fourth	21	34%	40	66%
	Fifth	4	27%	11	73%
	Sixth	10	40%	15	60%

**Table 4:** Stress Self-Assessment Questionnaire scores among undergraduate students in Saudi Arabia (*n* = 335).

No	Items	Mean	SD
1	Am I emotionally sensitive to comments or criticism from others	2.06	1.62
2	Am I quick to get angry or irritable	2.42	1.68
3	Am I an idealist, and tend to be dissatisfied with what I have done or what others have done	1.82	1.60
4	I suffer from a rapid heartbeat, sweating, and muscle tremors, such as those that occur in my face or body in general	1.16	1.57
5	Feel tension in my muscles? I feel tightness in my jaw, face, or body in general	1.37	1.68
6	I have problems sleeping	2.01	1.68
7	Feeling worried? I worry a lot	2.69	1.96
8	I suffer from physical symptoms such as digestive problems, pain, headaches, allergies or eczema	1.53	1.93
9	Feeling tired	2.49	1.87
10	Do I suffer from more serious health problems such as stomach ulcers, skin diseases, cholesterol problems, high blood pressure, or heart disorders	0.46	1.22
11	Smoke to motivate me or calm me down? Do I use products or medications for this purpose	0.37	1.04
	Total (sum score)	18.37	11.06

**Table 5:** Distribution of stress categories among undergraduate students in Saudi Arabia (*n* = 335).

No	Stress Level	*n*	Percentage
**1**	No	145	43.3
**2**	Low	150	44.8
**3**	Moderate	35	10.4
**4**	High	5	1.5

**Table 6:** Assessment of sleep quality among Undergraduate Students in Saudi Arabia (*n* = 335).

No	Component	Mean	SD
1	Subjective sleep quality	1.08	0.66
2	Sleep latency	1.64	0.97
3	Sleep duration	1.14	0.95
4	Sleep efficiency	0.79	1.15
5	Sleep disturbance	1.30	0.56
6	Use of sleep medication	0.21	0.64
7	Daytime dysfunction	1.47	0.91
	PSQI	7.62	3.11

**Table 7:** Distribution of sleep quality level among Undergraduate Students in Saudi Arabia (*n* = 335).

No	Sleep Quality	*n*	Percentage
1	Poor	247	73.7
2	Good	88	26.3

**Table 8:** Association between BRUXiq screening status and categorical variables (*n* = 335).

Factor	Category	Negative BRUXiq Screen *n* (%)	Positive BRUXiq Screen *n* (%)	χ^2^	*p*-Value	Effect Size (95% CI)
Stress level	No	87 (60.0%)	58 (40.0%)	51.217	<0.001	Cramér’s V = 0.391 (0.319–0.476)
	Low	49 (32.7%)	101 (67.3%)			
	Moderate	0 (0.0%)	35 (100.0%)			
	High	1 (20.0%)	4 (80.0%)			
Sleep quality	Poor	84 (34.0%)	163 (66.0%)	18.453	<0.001	φ = 0.235 (0.126–0.343)
	Good	53 (60.2%)	35 (39.8%)			
Academic year	First	46 (38.7%)	73 (61.3%)	5.641	0.343	Cramér’s V = 0.130 (0.038–0.222)
	Second	33 (47.1%)	37 (52.9%)			
	Third	23 (51.1%)	22 (48.9%)			
	Fourth	21 (34.4%)	40 (65.6%)			
	Fifth	4 (26.7%)	11 (73.3%)			
	Sixth	10 (40.0%)	15 (60.0%)			
Smoking	No	131 (41.9%)	182 (58.1%)	1.808	0.179	φ = 0.073 (0.003–0.173)
	Yes	6 (27.3%)	16 (72.7%)			

**Table 9:** Normality test (*n* = 335).

Variables	Bruxism	Kolmogorov-Smirnova	*p*-Value
Stress score	Negative BRUXiq screen	0.122	<0.001
	Positive BRUXiq screen	0.102	<0.001
Sleep quality score	Negative BRUXiq screen	0.056	0.200
	Positive BRUXiq screen	0.077	0.007

**Table 10:** Comparison of stress and sleep-quality scores according to BRUXiq screening status using the Mann–Whitney U test (values shown for groups are mean ranks; *n* = 335).

Bruxism Status	Stress	Sleep Quality
Negative BRUXiq screen	114.72	125.40
Positive BRUXiq screen	204.86	197.47
U	6264.0	7727
Z	−8.379	−6.731
*p*-value	<0.001	<0.001
Rank-biserial correlation (95% CI)	0.538 (0.437–0.634)	0.430 (0.315–0.534)

**Table 11:** Correlation Analysis between bruxism score and stress/sleep quality (*n* = 335).

Scales		BRUXiq Total Score	95%CI
Stress score	ρ	0.579	(0.503–0.646)
	*p*	<0.001	
Sleep quality score	ρ	0.408	(0.312–0.496)
	*p*	<0.001	

**Table 12:** Multivariable logistic regression of factors associated with a positive BRUXiq screen (*n* = 335).

Model Summary	Nagelkerke R-Squared	Chi-Square	*p*-Value	
	0.362	8.194	0.415	
Variables	Exp (B)	*p*-value	95%LCL	95%UCL
Stress	1.123	<0.001	1.083	1.165
Sleep quality	1.188	<0.001	1.074	1.314
Smoking	2.223	0.278	0.524	9.421
stimulant use	0.746	0.112	0.521	1.07
Physical activity				
Low	0.743	0.564	0.271	2.036
Moderate	0.461	0.127	0.17	1.246
Academic year				
First	1.191	0.731	0.44	3.22
Second	0.953	0.928	0.333	2.723
Third	0.555	0.308	0.179	1.723
Fourth	1.226	0.708	0.422	3.566
Fifth	1.557	0.617	0.275	8.807

**Table 13:** Roommate/bed-partner status and partner-observed sleep behaviors.

**A. Roommate/Bed-Partner Status (*n* = 335)**	***n***	**%**			
No bed partner or roommate	174	51.9			
Partner/roommate in another room	16	4.8			
Same room, not same bed	134	40.0			
Same bed	11	3.3			
**B. Partner-Observed Sleep Behavior (*n* = 161)**	**Not During Past Month**		**<1/Week**	**1–2/Week**	**≥3/Week**
Loud snoring	117 (73%)		26 (16%)	10 (6%)	8 (5%)
Breathing pauses	126 (78%)		21 (13%)	8 (5%)	6 (4%)
Leg twitching/jerking	74 (46%)		39 (24%)	31 (19%)	17 (11%)

**Table 14:** Sleep-disturbance themes reported by participants who provided open-ended responses (*n* = 60).

No	Disturbance	*n*	Percentage
1	Insomnia (difficulty falling or staying asleep)	10	16.7
2	Nightmares/bad dreams/scary dreams	9	15
3	Shortness of breath/difficulty breathing during sleep	8	13.3
4	Anxiety, overthinking, stress before or during sleep	8	13.3
5	Frequent waking up/interrupted sleep	7	11.7
6	Pain during or after sleep (joint, back, neck, jaw, etc.)	6	10.0
7	Sudden jolts/body jerks/panic upon falling asleep	4	6.7
8	Palpitations/fast heartbeat during sleep	4	6.7
9	Sleep paralysis/awareness during dreams/lucid dreams	4	6.7
10	Excessive movement/discomfort during sleep	3	5.0
11	Sweating during sleep	2	3.3
12	Numbness or gripping of hands during sleep	2	3.3
13	Crying/sadness/emotional distress after waking up	2	3.3
14	Thirst or waking to drink water	2	3.3
15	Difficulty maintaining sleep posture/discomfort	2	3.3
16	Mouth wetness or drooling during sleep	1	1.7
17	Light sleeping/waking up easily (e.g., when someone enters)	1	1.7
18	Sleep disturbances due to academic or life stress	2	3.3
19	Comments on the questionnaire (not sleep-related)	1	1.7

Note: Participants could report more than one sleep-disturbance theme; therefore, 78 themes were coded from 60 respondents and percentages do not sum to 100%.

## Data Availability

The datasets generated and/or analyzed during the current study are not publicly available due to the inclusion of confidential participant information. However, anonymized data may be made available by the corresponding author on reasonable request.
